# Morphological and Fitness Attributes of Young Male Portuguese Basketball Players: Normative Values According to Chronological Age and Years From Peak Height Velocity

**DOI:** 10.3389/fspor.2021.629453

**Published:** 2021-06-10

**Authors:** Sérgio Antunes Ramos, Luis Miguel Massuça, Anna Volossovitch, António Paulo Ferreira, Isabel Fragoso

**Affiliations:** ^1^CIDEFES, Faculty of Physical Education and Sport, Lusófona University, Lisbon, Portugal; ^2^CIPER, Faculdade de Motricidade Humana, SpertLab, Universidade de Lisboa, Lisbon, Portugal; ^3^ICPOL, Higher Institute of Police Sciences and Homeland Security, Lisbon, Portugal

**Keywords:** reference values, body size, velocity, change of direction ability, strength, talent programs

## Abstract

The aims of the present study were: (i) to describe the structural and functional attributes of young male Portuguese basketball players aged 12–16 years and (ii) to generate normative data according to chronological age and years from peak height velocity. A total of 281 male Portuguese young basketball players between the ages of 12 and 16 years were assessed in this study. Chronological age, maturity parameters (maturity offset and predicted age at peak height velocity), morphological (body mass, height, and skinfolds and length), and fitness (sprint, change of direction ability, jump, and upper body strength) attributes were measured. Descriptive statistics were determined for the age and maturity status, and the 10th, 25th, 50th, 75th, and 90th percentiles were chosen as reference values. Descriptive and normative values of the players' morphological and fitness attributes, stratified by age and maturity status, are provided. The normative values of age at peak height velocity (category YAPHV = 0) showed that regional basketball players presented average values (50th percentile) of 169.8 cm for height, 173.3 cm for arm span, 55.6 kg for body mass, 3.34 s for the 20-m speed test, 10.31 s for the *T*-test, 4.75 m for the 2-kg medicine ball throw, 66.9 kg for the combined right and left handgrip strength, and 30.1 and 35.9 cm for jump height in the countermovement jump (CMJ) and CMJ with arm swing, respectively. In conclusion, these results may be helpful to quantify and control an athlete's performance over time and to adjust strength and conditioning programs to biological demands.

## Introduction

Basketball is a complex team sport where physical attributes, physiological performance, technical skills, tactical knowledge, and psychological attributes contribute to the players' and overall team's success (Trninić and Dizdar, 2000; Ostojic et al., 2006; Drinkwater et al., 2008; Torres-Unda et al., 2013, 2016; Ramos et al., 2019, 2020). Due to the multifactorial nature of basketball performance, identifying and selecting young players with the potential to attain high levels of performance in adult age is a difficult task. The variables influencing performance at a young age may be different from those that influence the success of adult athletes (Baker and Wattie, 2018); as has been demonstrated in various sports, the earlier the sport selection takes place, the lower its accuracy (Vaeyens et al., 2009; Te Wierike et al., 2014). However, knowledge of which factors influence players' success at different age categories can be a valuable resource in talent selection and all the talent development process (Hoare, 2000; Torres-Unda et al., 2013).

Recognizing the difficulty of finding valid measures to evaluate a prospective young athlete's potential justifies the need to analyze the morphological and fitness profiles of successful young players at different stages of their development (Vaeyens et al., 2008; Johnston et al., 2018).

The appropriate planning of a long-term training process requires the use of control tests to keep up with the evolution of athletes' morphological and physical characteristics in relation to their specific sport performance (Kuzuhara et al., 2018; Ryan et al., 2018; Myburgh et al., 2020). It was shown that the monitoring of players' attributes is particularly important during periods of accelerated biological development in order to control the adaption to training exposure, to reduce the injury risk, and, thereby, to enhance the coaching effectiveness process (Ryan et al., 2018; Salter et al., 2021). In this regard, establishing normative data for a particular population may improve the sensitivity of the selection criteria (Ryan et al., 2018; Salter et al., 2021) and provide coaching staff with specific references for the athletes' development program by comparing the morphological and physical status of the assessed players with the age normative data for specific populations (Ocarino et al., 2021).

Although the age- and sex-specific physical fitness reference data for Portuguese children and adolescents aged 10–18 years have been presented (Santos et al., 2014), there are no normative data for specific populations of Portuguese young basketball players based on a representative sample. It would be useful to compare the available normative data for Portuguese adolescents with the reference values of the young basketball players' population.

Previous studies on young basketball players highlighted the relevance of anthropometric (i.e., height, body mass, and arm span) and functional (i.e., speed, agility, upper body strength, and jumping ability) attributes in young players' performance (Torres-Unda et al., 2013, 2016; Ramos et al., 2019, 2020). Ramos et al. (2020) found that players from the better-ranked teams were faster, more agile, and had more upper body explosive strength than players from lower-ranked teams. Another study found that height, agility, countermovement jump (CMJ) power, and handgrip strength were predictors of individual performance (evaluated by the Performance Index Rating) in under-14 (U-14) young basketball players (Ramos et al., 2019). In the same study, significant correlations were also identified between playing performance and the anthropometric attributes (i.e., body mass, stature, and arm span) of U-14 young basketball players, suggesting that taller and heavier players perform better in matches than their shorter and smaller peers. In addition, Torres-Unda et al. (2016) observed that players who performed better had longer body lengths and also that players with greater jump capability scored more points.

Furthermore, the variability on the maturation processes may lead to the different biological development levels of young basketball players found in the same chronological age, resulting in a higher physical ability and, consequently, to a superior basketball game performance of those athletes whose biological development was initiated earlier (Torres-Unda et al., 2013, 2016; Ramos et al., 2019, 2020). Previous studies have found differences in anthropometric and physiological capacities, and in biological age, among U-14 Spanish (Torres-Unda et al., 2013, 2016) and Portuguese (Ramos et al., 2019, 2020) male basketball players with different skill levels.

From the talent development perspective, it would be useful to have normative data of the physical attributes according to not only the chronological age but also the maturity status. These reference values can guide coaches' decisions during talent selection programs at different age categories and can also help to interpret better the results achieved by young players during their training process, thus contributing to talent development.

Although several studies on the morphological and physical attributes of Portuguese young basketball players have been conducted (Coelho e Silva et al., 2010; Guimarães et al., 2019a; Ramos et al., 2019, 2020), they did not provide comprehensive normative data, established according to age and maturity status. Furthermore, the results of the studies conducted with young Portuguese basketball players are somewhat contradictory. While some of them confirmed the significant influence of maturity status on physical performance (Arede et al., 2019; Guimarães et al., 2019b), others reported that functional capabilities were largely independent of maturity status, especially after controlling for variations in body size (Coelho e Silva et al., 2008). Therefore, the purpose of this study was two-fold: (i) to analyze the morphological and fitness attributes of young male Portuguese basketball players aged 12–16 years and (ii) to establish normative data according to the chronological age and maturity status of young players. Taking into consideration the influence of biological maturation on a player's individual performance (Torres-Unda et al., 2016; Ramos et al., 2019) and, consequently, on the selection process of young basketball players (Coelho e Silva et al., 2004; Ramos et al., 2019), the normative data will be established according to maturity status.

## Methods

### Subjects

In Portugal, the initial stages of the selection process start with the under-14's (U-14) category, when the most promising players aged 12–14 years are selected by the best clubs or regional teams. Every year, the Portuguese Basketball Federation organizes a national tournament, where the best of the country's U-14 and U-16 male regional teams compete for 5 days. Taking advantage of this event, a convenient sample of 281 male basketball players (mean ± SD = 14.51 ± 0.98 years) between the ages of 12 and 16 years was evaluated in this study. These participants represented the first division male regional selection teams that competed in the Portuguese Festival of Youth Basketball. In agreement with the Portuguese Basketball Federation, the supposed best U-14 (*n* = 173) and U-16 (*n* = 108) male Portuguese basketball players were tested during this tournament, over three consecutive seasons. This allowed us to create an extensive database of the morphological and fitness attributes of high-level young players according to their chronological age and years from age at peak height velocity (YAPHV).

All participants received a clear explanation of the aims and procedures of this study. Only the players whose parents or legal guardians have signed an informed consent form were permitted to participate in the study. The study was authorized by the Ethics Committee of the Faculty of Physical Education and Sport—Universidade Lusófona and was performed according to the Helsinki Declaration.

### Procedures

A mix longitudinal design was used for this study. Data related to players' practice experiences (i.e., years of basketball practice) and training load (i.e., hours of practice per week) were collected with a specific questionnaire. Data related to players' morphologic and fitness characteristics were collected by the researchers. The measurements took place on the first day of the tournament to avoid the influence of players' fatigue on the results of measurements. However, some players were measured after the competition had started. In these cases, it was guaranteed that the evaluations were conducted at least 2 h after the game had been played. The test batteries used in the study covered maturity status and morphological and fitness evaluations, which have already been described in detail in a previous paper (Ramos et al., 2020). Anthropometric tests were undertaken before the functional skills tests.

### Age and Maturity Status Evaluations

Chronological age (CA), in decimals (decimal age), was calculated subtracting the birth date from the observed date using the reference decimal age tables (Healy et al., 1981). The CA group was defined by the whole year (i.e., 12 years = 12.00–12.99 years, 13 years = 13.00–13.99 years, 14 years = 14.00–14.99 years, 15 years = 15.00–15.99 years, and 16 years = 16.00–16.99 years) (Ramirez-Velez et al., 2017).

The maturity offset (YAPHV) was predicted from a sex-specific equation (Mirwald et al., 2002) and provides the distance in years before or after the age at peak height velocity. The predicted age at peak height velocity (APHV) was calculated by subtracting the predicted maturity offset from the CA obtained at the time of observation (Mirwald et al., 2002). Chronologic age, stature, sitting height, and estimated leg length (stature minus sitting height) were used to predict the maturity offset and are described in *Morphological Evaluation*. Maturity group was defined with the whole year as the midpoint of the range [i.e., −1 = (−1.50, −0.51), 0 = (−0.50, 0.49), 1 = (0.50, 1.49), 2 = (1.50, 2.49), and 3 = (2.50, 3.49)] (Kalabiska et al., 2020).

All the equations used to predict YAPHV (maturity offset) or the APHV have the same major limitations (Malina and Kozieł, 2014; Malina et al., 2015). The advanced maturity status of male adolescent athletes and the relatively narrow range of variation at the predicted ages of peak height velocity (PHV) may influence the maturity status evaluation during adolescence and may impair its utility and effectiveness on talent identification and development programs when used at a particular moment. Recently, Rommers et al. (2020) have shown that none of the published equations provided an accurate prediction for individuals. However, although the stability of the predictions within individuals are poor and group classification is not exactly accurate, APHV predicted by the Mirwald equation (Mirwald et al., 2002) can be successfully applied among boys who are average (on time) in maturation and during the growth spurt period (~12–15 years). Moreover, Arede et al. (2020) validated the adult height prediction using a small number of unpublished Portuguese cases, according to Sherar et al. (2005). The authors showed an almost perfect relationship and a substantial agreement between the observed values of predicted adult height (PAH) and the estimated values of PAH. Additionally, adult height estimated through the formula of Sherar et al. (2005) was only slightly higher than the adult height estimated by the method of Khamis and Roche (1994). These results reinforce the possible use of the Mirwald equation (Mirwald et al., 2002) even when PAH measures are being considered. Taking into consideration the importance of biological maturity for talent identification, and the difficulties of implementing maturity protocols other than the above-mentioned, the use of references organized by maturity groups, according to Mirwald et al. (2002), and obtained from a sample that supposedly gathers the best U-14 and U-16 male Portuguese basketball players, could provide insights into the sport-specific skills necessary to be ranked among the best national players in each age and maturity group (Huijgen et al., 2010) and may aid coaches to identify young players potentially at risk (Costa e Silva et al., 2017).

### Morphological Evaluation

Body mass, stature, sitting height, and three skinfolds—triceps (Caterisano et al., 1997), calf (GML), and subscapular (SBS), were measured according to the International Society for the Advancement of Kinanthropometry guidelines (Marfell-Jones et al., 2006). Arm span and hand span were also measured (Massuça and Fragoso, 2013). Body mass was measured with a Secca body scale (model 761 7019009) to the nearest 0.5 kg, and stature and sitting height were measured with a Siber-Hegner anthropometric kit to the nearest of 0.1 cm. All measurements were made by an ISAK anthropometric technician who holds a level 2 qualification. The intra-observer technical errors of measurements, %TEM (and the coefficient of reliability, *R*), were well below the accepted maximum for stature (*R* ≥ 0.98), 5% for skinfolds (*R* = 0.90–0.98), and 1% for breadths and girths (*R* = 0.92–0.98) (Marfell-Jones et al., 2006). The body composition analysis included the evaluation of relative fat mass (%FM) and absolute free-fat mass (FFM, in kilograms), estimated from the skinfold values. The %FM was calculated as the arithmetic mean of the %FM values obtained through the equations proposed by Lohman [equation 1: %FM = 1.35 × (TRI + SBS) – 0.012 × (TRI + SBS)^2^ – I, where *I* is constant] (Lohman, 1986) and Slaughter et al. [equation 2: %FM = 0.735 × (TRI + GML) + 1] (Slaughter et al., 1988). The body mass index (BMI) was calculated using the formula, BMI = Body mass/Stature^2^ (in kilograms per square meter).

### Fitness Evaluation

Before the fitness tests, all participants performed a standard 20-min warm-up routine (slow jogging followed by static and dynamic stretching) supervised by the researchers. The players were allowed a 10-min passive rest between tests, as well as water breaks and extra rest time. Each participant was verbally instructed and encouraged to give his/her maximum effort. Three trials were given for each test. The first was a practice trial for the familiarization with the test; the second and third trials were retained for analysis. All players completed seven fitness tests, from which nine variables were collected for analysis. The established order of physical tests allowed avoiding performing two consecutive tests for the upper or the lower body. In each team, due to competition constraints, the players were divided into groups of four elements and went through the established order. All data were collected by the researchers.

#### Speed Test

The 20-m speed test was performed and consisted of a 20-m linear sprint effort (Jakovljevic et al., 2012). The time of the speed test was recorded in seconds and hundredths of a second using photoelectric cells (Wireless Sprint system, Brower Timing Systems, Salt Lake City, UT, USA), and the best time of two attempts was registered.

#### T-Test

A *T*-test was used for the change of direction (COD) ability assessment (Delextrat and Cohen, 2009; Jakovljevic et al., 2012). The time was recorded in seconds and hundredths of a second using photoelectric cells (Wireless Sprint system, Brower Timing Systems, Salt Lake City, UT, USA), and the best time of two attempts was registered.

#### Jump Tests

The vertical jumping ability was tested using the CMJ and countermovement jump with arm swing (CMJ-S) (Bosco et al., 1983). The height (in centimeters) and the relative power (in watts per kilogram) of vertical jumps were recorded with a Chronojump measurement technology (Bosco System, Globus, Italy). The best of two attempts was considered.

#### Two-Kilogram Medicine Ball Throw

The upper limb explosive strength was tested using the 2-kg medicine ball throw (MBT) (Delextrat and Cohen, 2009). Participants started the test from a sitting position with the back against the wall using a release from the chest. The distance (in centimeters) attained as the best of two attempts was recorded.

#### Handgrip Strength

Handgrip (HG) strength was assessed with a handgrip test using a dynamometer (Takei Physical Fitness Test, TKK 5001, GRIP-A) (España-Romero et al., 2010). Subjects performed the test twice with each hand, and the sum of the best results achieved by each left and right hand was considered (in kilograms).

#### Sit and Reach Test

Flexibility was assessed using the sit and reach test (Mayorga-Vega et al., 2014). Each subject was seated barefoot on the floor with legs out straight ahead and with their feet placed with the soles flat against the sit and reach box. With hands on top of each other and palms facing down, each player tried to reach forward along the measuring line as far as possible. The score of the test was recorded to the nearest centimeter as the distance reached by the tip of the fingers. The vertical line of the feet soles was considered as a plane counted as 0 cm. Negative and positive centimeters were considered when the players reached forward, respectively, before and after this vertical plane.

### Statistical Analyses

All the analyses were performed using the Statistical Package for the Social Sciences (SPSS, version 22.0, IBM SPSS, Chicago, IL, USA). The normality of the variables was assessed with the Kolmogorov–Smirnov test, and the equality of the variances was established with Levene's test. Intraclass correlation coefficients (Toong et al., 2018) were calculated for the 20-m speed test, *T*-test, jump tests (i.e., the CMJ and CMJ-S height and relative power), 2-kg MBT test, HG test, and the sit and reach test (see Table 1). The normality and equality of variances have been checked and confirmed for all variables, and the descriptive statistics (mean and standard deviation) were determined for each age (e.g., 13 years = 13.00–13.99 years) and maturity (e.g., YAPHV) group. Subjects were divided into percentiles, and the 10th, 25th, 50th, 75th, and 90th percentiles were considered as the reference values for each age and maturity group. Complementarily, the 25th, 50th, and 75th percentiles were presented graphically (see figures). Graphs were generated with GraphPad Prism 8.0 (GraphPad Software, Inc., San Diego, CA, USA).

**Table 1:** Intraclass correlation statistics for inter-rater reliability for the physical tests.

	**ICC^**a**^**	**95% CI**		***F*** **test with true value 0**			
		**Lower bound**	**Upper bound**	**Value**	**df1**	**df2**	**Sig**.
20-m speed test	0.937	0.921	0.949	30.514	287	287	<0.001
*T*-test	0.946	0.929	0.959	35.969	197	197	<0.001
CMJ heigth	0.962	0.951	0.969	49.917	287	287	<0.001
CMJ relative power	0.992	0.990	0.994	248.466	287	287	<0.001
CMJ-S height	0.958	0.947	0.966	46.393	287	287	<0.001
CMJ-S relative power	0.980	0.975	0.984	97.796	287	287	<0.001
2-kg MBT test	0.968	0.960	0.974	60.926	294	294	<0.001
HG rigth hand	0.980	0.975	0.984	100.302	292	292	<0.001
HG left hand	0.980	0.975	0.984	99.004	292	292	<0.001
Sit and reach test	0.922	0.903	0.938	24.769	289	289	<0.001

a *ICC estimates and their 95% confidence intervals were calculated using SPSS statistical package version 22.0 based on a consistency, two-way random-effect model. CI, Confidence interval; CMJ, Countermovement jump; CMJ-S, Countermovement jump with arms swing; ICC, Intraclass correlation; HG, handgrip; MBT, Medicine ball throw*.

## Results

Maturational parameters and the morphological and fitness attributes of U-14 and U-16 young male basketball players who participated in the Portuguese National Basketball Championship for regional selection teams are presented in Table 2.

**Table 2:** Descriptive statistics (mean ± SD) for training experience, maturational parameters, morphological, and fitness characteristics of U-14 and U-16 male players participated in the U-14 Portuguese Basketball Championship for regional teams.

	**U-14 category (*n* = 173)**	**U-16 Category (*n* = 108)**
Practice experience (years)	5.2 ± 2.5	6.7 ± 2.5
Training load (hrs-week^−1^)	6.1 ± 1.6	6.8 ± 2.5
CA (years)	13.8 ± 0.4	15.7 ± 0.4
Maturity offset (years)	0.54 ± 0.7	2.04 ± 0.6
APHV (years)	13.3 ± 0.6	13.6 ± 0.6
**Morphology**		
Body mass (kg)	60.1 ± 9.9	68.8 ± 9.8
Stature (cm)	173.5 ± 8.4	180.5 ± 7.3
Arm span (cm)	176.4 ± 9.5	186.7 ± 8.6
Hand span (cm)	22.1 ± 1.6	22.6 ± 1.4
BMI (kg/m^2^)	19.9 ± 2.2	21.0 ± 2.3
%FM	16.5 ± 5.4	13.7 ± 4.7
Fat-free mass (kg)	49.7 ± 6.7	58.9 ± 7.1
**Fitness**		
V20-m (s)	3.35 ± 0.22	3.12 ± 0.11
TT (s)	10.35 ± 0.60	9.55 ± 0.50
SUM HG (kg)	69.2 ± 15.7	85.9 ± 15.7
MBT (m)	4.91 ± 0.8	6.13 ± 0.7
CMJ Height (cm)	30.4 ± 4.8	34.4 ± 4.6
CMJ Power (W)	719 ± 143	891 ± 128
CMJ-S Height (cm)	35.9 ± 5.6	41.7 ± 5.5
CMJ-S Power (W)	781 ± 154	982 ± 141
Sit and reach (cm)	−1.3 ± 7.7	4.0 ± 9.7

*APHV, age at peak height velocity; BMI, body mass index; CA, chronological age; CMJ, countermovement jump; CMJ-S, countermovement jump with arm swing; MBT, medicine ball throw; SUM HG, sum of right and left handgrip; TT, T-test; V20-m, 20 meters speed test; YAPHV, years from age at peak height velocity; %FM, Fat mass percentage*.

**Figure 1:**
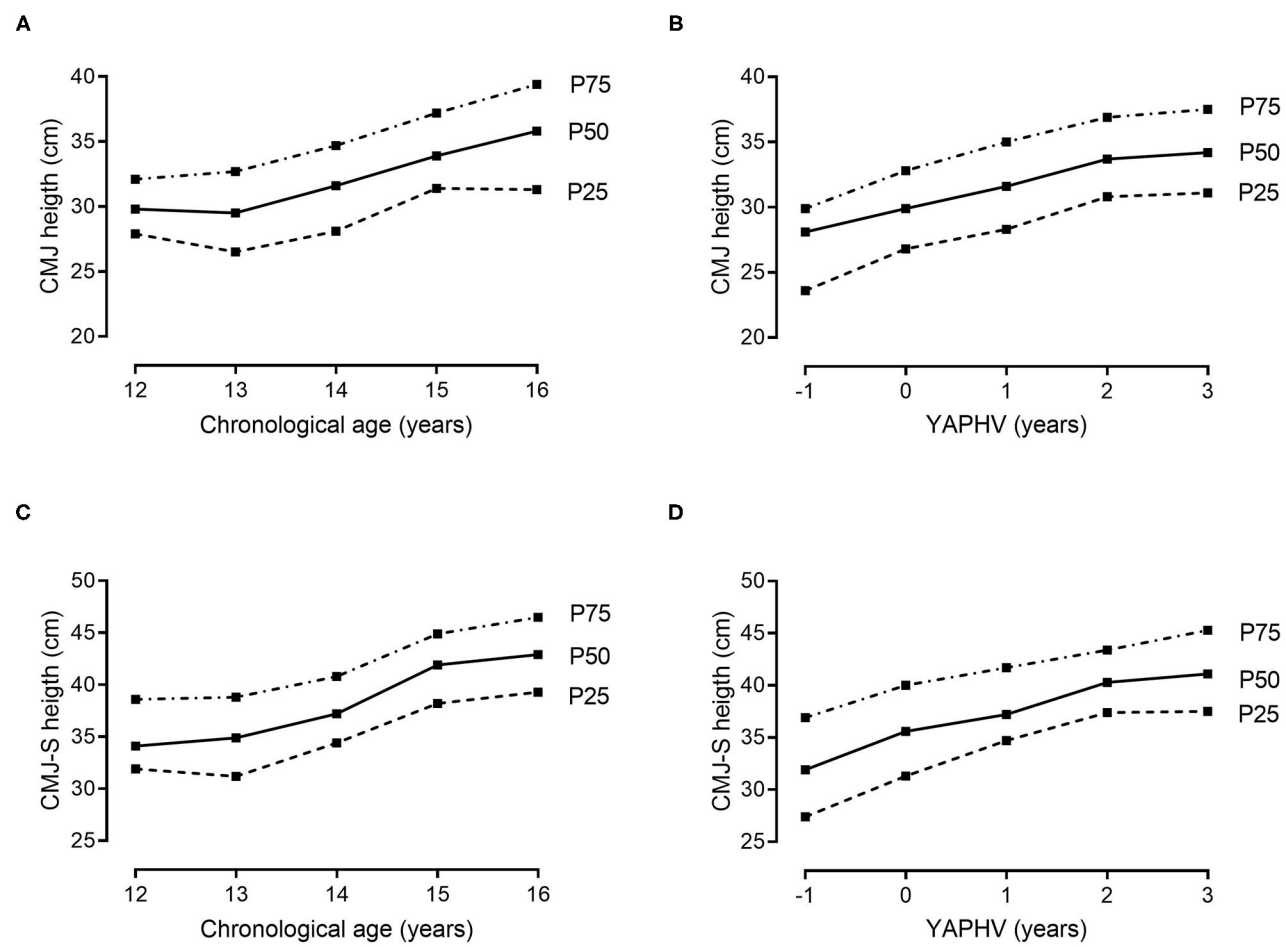
References values (10th, 25th, 50th, 75th and 90th percentiles) for body mass, stature and arm span of young Portuguese male basketball players, according to their chronological age and maturity status.

**Figure 2:**
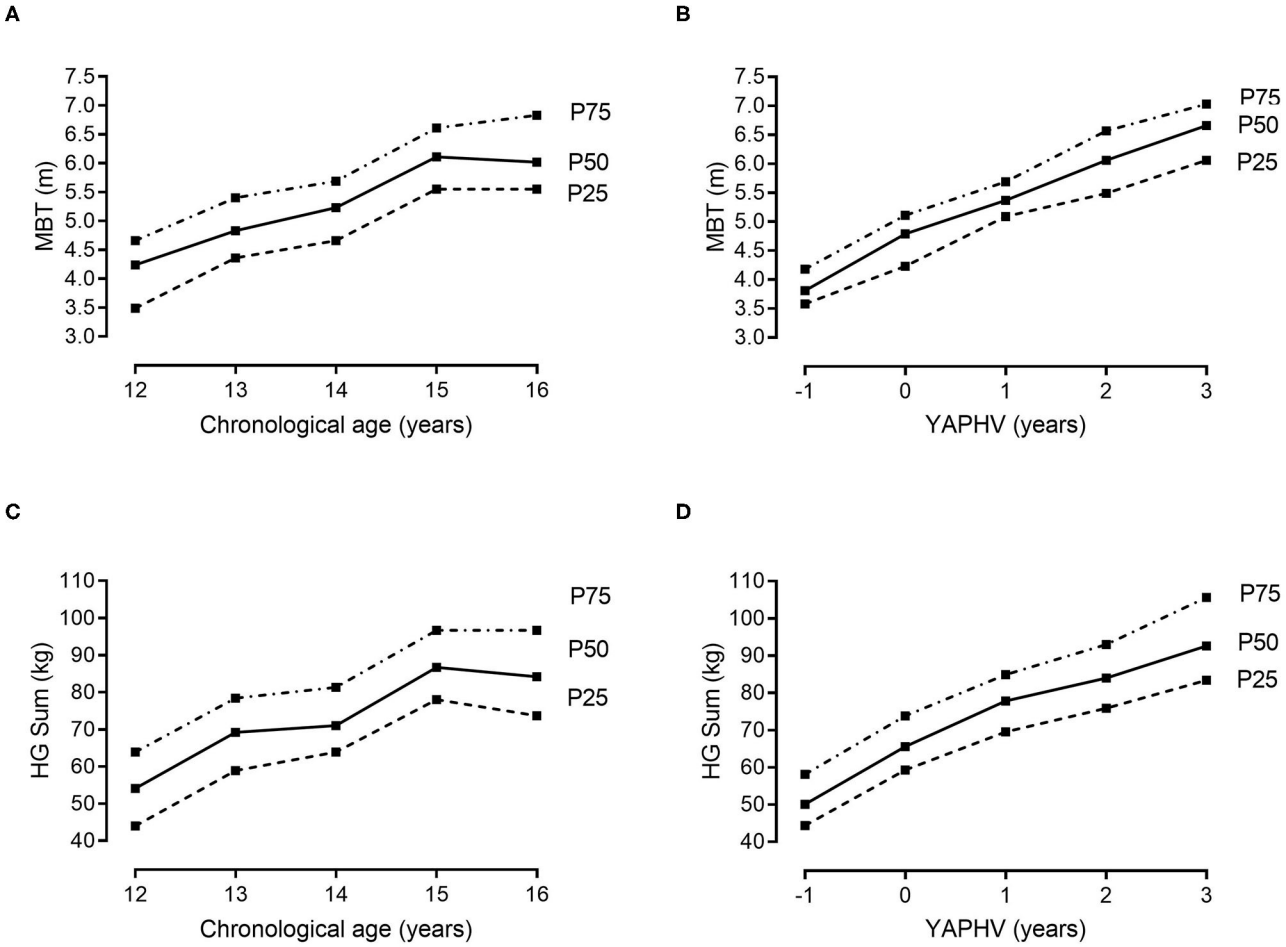
References values (10th, 25th, 50th, 75th and 90th percentiles) for speed and agility of young Portuguese male basketball players, according to their chronological age and maturity status.

**Figure 3:**
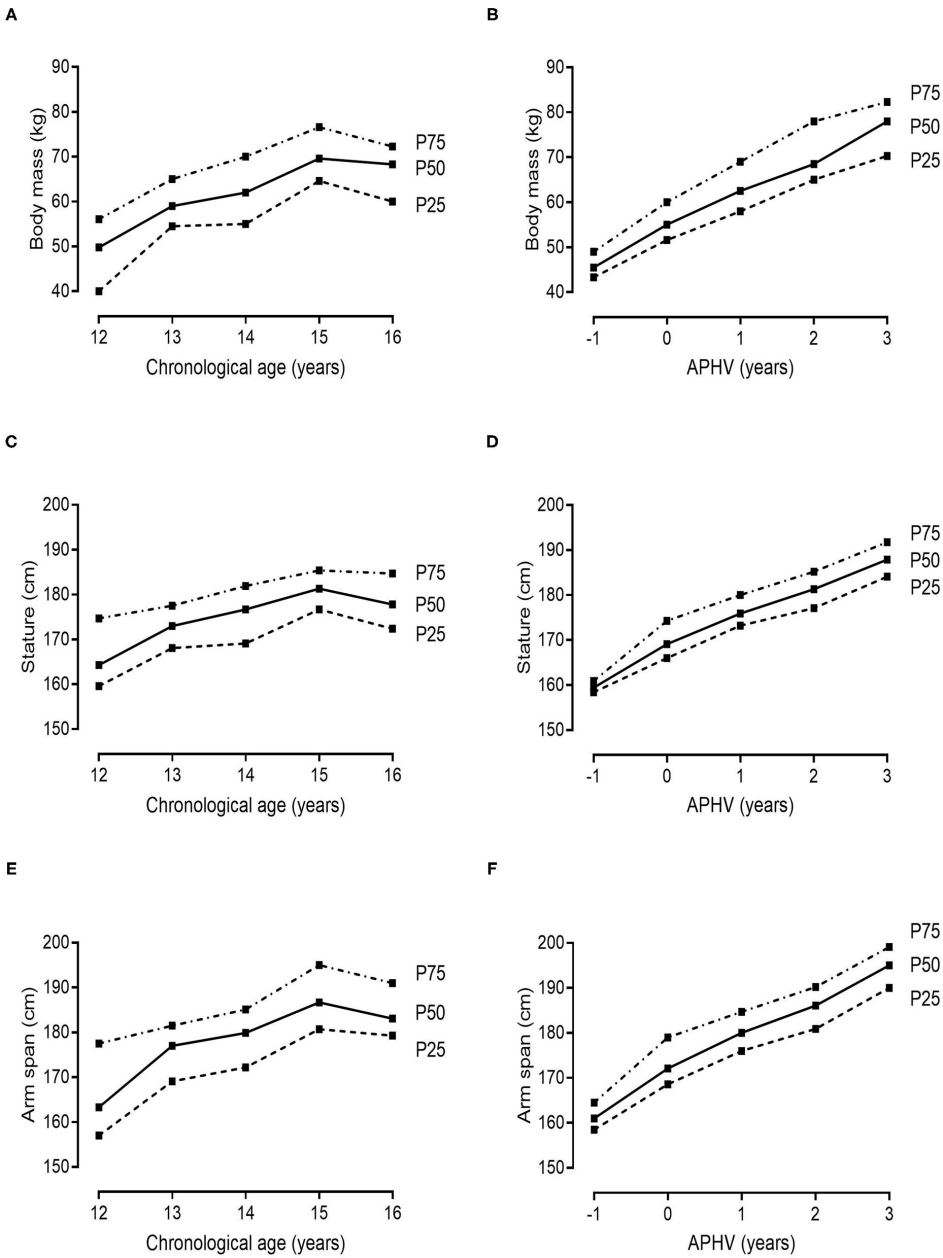
References values (10th, 25th, 50th, 75th and 90th percentiles) for CMJ and CMJ-S height of young Portuguese male basketball players, according to their chronological age and maturity status.

**Figure 4:**
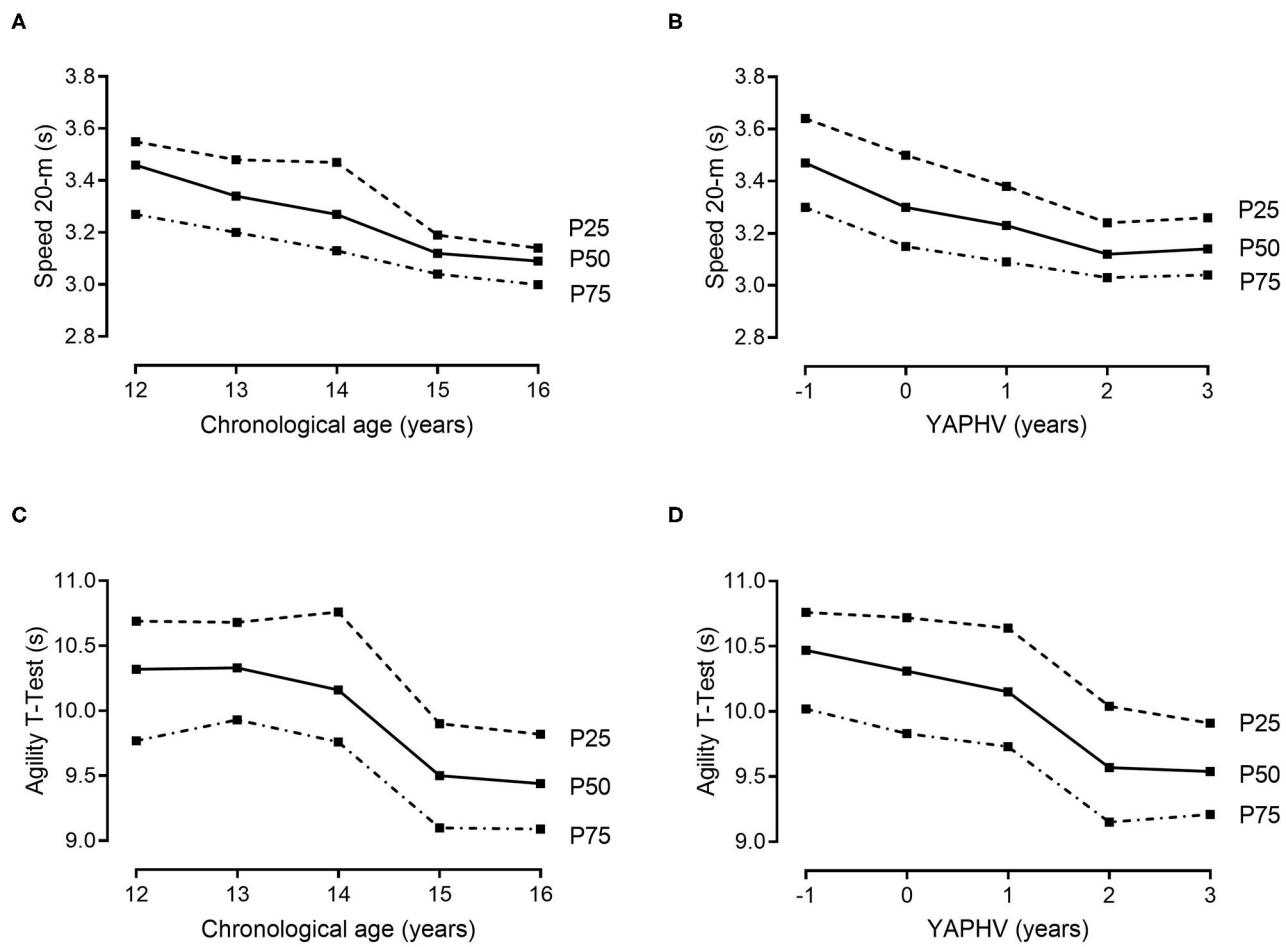
References values (10th, 25th, 50th, 75th and 90th percentiles) for MBT and HG strength of young Portuguese male basketball players, according to their chronological age and maturity status.

Descriptive and normative values for the players' morphological and fitness attributes, stratified by chronological age and YAPHV, are provided in Table 3, 4, respectively. Complementarily, the 25th, 50th, and 75th percentiles of (i) body size and arm span, (ii) speed and COD ability, (iii) countermovement jumps, and (iv) the 2-kg MBT and HG strength are graphically presented in Figures 1–4, respectively.

**Table 3:** Descriptive statistics (mean ± SD) and reference values (10th, 25th, 50th, 75th, and 90th percentiles) for morphology of young Portuguese male basketball players, according to their chronological age and maturity status.

	**Chronological age (years)**					**YAPHV**				
		**12**	**13**	**14**	**15**	**16**	**−1**	**0**	**1**	**2**	**3**
	***N***	**12**	**97**	**75**	**70**	**27**	**18**	**62**	**102**	**71**	**28**
Body Mass (kg)	M ± SD	49.0 ± 10.0	59.7 ± 8.9	62.2 ± 10.4	69.9 ± 9.9	66.8 ± 9.4	46.1 ± 6.3	55.5 ± 5.2	64.1 ± 7.8	68.4 ± 6.7	77.7 ± 9.0
	P10	34.0	46.0	49.3	57.9	51.7	35.8	47.8	55.3	59.7	66.9
	P25	40.0	54.5	55.0	64.6	60.0	43.3	51.3	58.0	65.0	70.3
	P50	49.8	59.0	62.0	69.6	68.3	45.5	55.0	62.5	68.5	78.0
	P75	56.1	65.0	70.0	76.6	72.3	49.0	60.0	69.0	72.0	82.3
	P90	–	70.5	75.0	82.3	80.8	56.6	65.0	75.0	77.9	88.9
Height (cm)	M ± SD	165.9 ± 8.8	172.6 ± 8.1	176.2 ± 8.1	181.4 ± 6.9	179.1 ± 8.1	159.6 ± 3.2	169.8 ± 5.2	176.6 ± 5.4	180.9 ± 5.7	188.0 ± 5.5
	P10	154.5	160.4	165.3	172.3	169.1	154.2	162.8	169.3	172.7	181.5
	P25	159.0	168.1	169.1	176.7	172.4	158.4	166.0	173.2	177.0	184.1
	P50	164.3	173.0	176.7	181.3	177.8	159.4	169.0	175.9	181.3	187.9
	P75	174.7	177.5	181.9	185.4	184.7	160.9	174.1	180.0	185.2	191.8
	P90	–	184.0	186.2	190.3	191.1	163.1	176.0	184.3	189.1	196.5
Arm span (cm)	M ± SD	168.9 ± 11.9	175.8 ± 9.2	179.2 ± 9.7	187.5 ± 8.1	185.5 ± 9.4	161.6 ± 4.6	173.2 ± 7.4	180.3 ± 7.2	186.1 ± 6.5	194.3 ± 7.1
	P10	154.3	160.9	162.3	175.9	173.6	154.8	162.8	170.3	176.4	183.9
	P25	157.0	169.1	172.2	180.7	179.3	158.5	168.3	176.0	180.9	190.0
	P50	163.3	177.0	179.9	186.7	183.1	161.0	172.0	180.0	186.1	195.0
	P75	177.5	181.5	185.1	195.0	191.0	164.5	179.0	184.7	190.2	199.1
	P90	–	187.0	189.5	197.8	199.7	168.2	182.0	188.9	196.0	204.4
Hand span (cm)	M ± SD	21.2 ± 1.5	22.0 ± 1.6	22.5 ± 1.5	22.7 ± 1.5	22.5 ± 1.2	20.1 ± 1.6	21.8 ± 1.4	22.6 ± 1.2	22.7 ± 1.4	23.1 ± 1.4
	P10	19.6	20.2	20.7	21.0	20.9	18.0	20.4	21.2	20.7	20.9
	P25	20.0	21.0	21.5	21.5	21.6	18.6	21.0	21.6	21.7	22.1
	P50	20.9	22.0	22.6	22.6	22.6	20.3	21.9	22.6	22.8	23.2
	P75	22.9	23.1	23.3	24.0	23.5	21.4	22.5	23.5	23.5	24.0
	P90	–	23.9	24.3	24.7	23.6	22.3	23.4	24.4	24.3	25.1
BMI (kg/m^2^)	M ± SD	17.6 ± 2.1	20.0 ± 2.0	20.0 ± 2.3	21.2 ± 2.4	20.8 ± 2.0	18.1 ± 2.3	19.3 ± 1.7	20.5 ± 2.3	20.9 ± 1.8	22.0 ± 2.6
	P10	14.2	17.7	16.6	18.7	17.6	14.3	17.1	18.0	19.1	18.9
	P25	15.6	18.8	18.6	20.0	19.6	16.9	18.1	19.1	20.0	20.2
	P50	17.8	19.8	20.1	20.8	20.8	17.7	19.3	20.3	20.7	21.9
	P75	19.4	20.9	21.8	22.5	22.7	19.4	20.3	21.8	22.0	23.2
	P90	–	22.4	22.7	24.1	23.3	21.8	21.4	22.9	22.8	25.2
%FM	M ± SD	13.2 ± 3.0	16.7 ± 5.4	16.1 ± 5.3	14.1 ± 5.2	13.2 ± 3.5	15.5 ± 7.6	15.3 ± 4.4	17.0 ± 5.7	14.4 ± 4.6	14.7 ± 5.6
	P10	9.5	11.6	10.0	9.2	8.8	6.3	10.3	9.9	9.2	9.2
	P25	10.7	13.4	12.4	9.7	10.1	10.5	12.6	13.3	11.4	10.2
	P50	12.4	15.3	14.8	13.2	12.5	13.9	14.2	16.2	13.2	14.0
	P75	15.8	18.9	20.0	16.9	15.8	19.5	18.0	20.1	17.1	17.3
	P90	–	25.2	24.0	22.2	18.6	26.1	22.1	24.3	22.1	22.2
Fat-free mass (kg)	M ± SD	42.0 ± 7.9	49.3 ± 6.2	51.7 ± 6.9	59.8 ± 7.2	57.7 ± 6.7	38.0 ± 3.3	46.9 ± 4.2	52.9 ± 4.6	58.4 ± 4.6	65.8 ± 5.1
	P10	30.2	40.2	43.7	50.1	47.8	33.4	40.9	47.1	52.5	58.4
	P25	35.3	46.2	47.2	55.1	53.4	35.6	44.2	49.5	55.6	62.6
	P50	41.7	49.8	51.7	59.6	58.1	38.2	47.2	52.3	58.7	66.0
	P75	48.0	53.7	56.6	64.2	63.2	41.1	49.4	55.4	60.9	70.1
	P90	–	57.7	60.4	70.8	68.4	42.3	52.2	58.4	63.2	73.2

*BMI, body mass index; YAPHV, years from age at peak height velocity; %FM, relative fat mass*.

**Table 4:** Descriptive statistics (mean ± SD) and reference values (10th, 25th, 50th, 75th, and 90th percentiles) for fitness of young Portuguese male basketball players, according to their age and maturity status.

	**Chronological age (years)**					**YAPHV**				
		**12**	**13**	**14**	**15**	**16**	**−1**	**0**	**1**	**2**	**3**
	***N***	**12**	**97**	**75**	**70**	**27**	**18**	**62**	**102**	**71**	**28**
Speed 20-m (s)	M ± SD	3.42 ± 0.19	3.35 ± 0.22	3.30 ± 0.23	3.13 ± 0.12	3.09 ± 0.11	3.42 ± 0.21	3.34 ± 0.25	3.26 ± 0.19	3.14 ± 0.17	3.15 ± 0.13
	P10	–	3.66	3.61	3.31	3.24	3.67	3.69	3.52	3.38	3.31
	P25	3.55	3.48	3.47	3.19	3.14	3.64	3.50	3.38	3.24	3.26
	P50	3.46	3.34	3.27	3.12	3.09	3.47	3.30	3.23	3.12	3.14
	P75	3.27	3.20	3.13	3.04	3.00	3.30	3.15	3.09	3.03	3.04
	P90	3.10	3.06	3.00	3.00	2.91	3.06	3.04	3.03	2.95	2.97
*T*-Test (s)	M ± SD	10.25 ± 0.47	10.35 ± 0.57	10.28 ± 0.68	9.56 ± 0.49	9.50 ± 0.53	10.54 ± 0.79	10.31 ± 0.63	10.21 ± 0.61	9.67 ± 0.66	9.61 ± 0.53
	P10	–	11.20	11.33	10.16	10.27	11.84	11.21	11.10	10.69	10.46
	P25	10.69	10.68	10.76	9.90	9.82	10.76	10.72	10.64	10.04	9.91
	P50	10.32	10.33	10.16	9.50	9.44	10.47	10.31	10.15	9.57	9.54
	P75	9.77	9.93	9.76	9.10	9.09	10.02	9.83	9.73	9.15	9.21
	P90	9.43	9.62	9.43	9.03	8.84	9.57	9.57	9.47	8.94	9.02
Sum HG (kg)	M ± SD	56.4 ± 18.1	69.1 ± 15.9	71.9 ± 14.0	87.7 ± 13.2	86.2 ± 13.1	52.5 ± 10.3	66.9 ± 12.2	78.1 ± 12.3	83.7 ± 16.3	93.4 ± 12.4
	P10	39.5	51.6	56.9	70.6	68.9	41.2	51.06	63.9	68.1	77.3
	P25	44.0	58.9	63.9	78.0	73.7	44.8	59.3	69.5	76.0	83.4
	P50	54.1	69.2	71.0	86.7	84.2	50.1	65.6	77.8	84.1	92.6
	P75	63.9	78.4	81.3	97.7	96.7	57.7	73.8	84.9	93.0	105.2
	P90	–	91.9	90.4	108.6	107.2	71.6	83.9	95.6	101.7	109.6
MBT (m)	M ± SD	4.25 ± 0.92	4.84 ± 0.72	5.19 ± 0.84	6.13 ± 0.62	6.13 ± 0.72	3.97 ± 0.55	4.75 ± 0.60	5.39 ± 0.55	6.05 ± 0.64	6.49 ± 0.70
	P10	3.30	3.88	4.17	5,31	5.23	3.37	4.09	4.67	5.24	5.39
	P25	3.49	4.36	4.66	5.55	5.55	3.51	4.23	5.09	5.51	6.06
	P50	4.24	4.83	5.23	6.11	6.02	3.75	4.79	5.37	6.02	6.66
	P75	4.66	5.40	5.69	6.61	6.83	4.15	5.11	5.69	6.56	7.03
	P90	–	5.80	6.44	7.04	7.07	4.88	5.73	6.14	6.91	7.28
CMJ height (cm)	M ± SD	30.2 ± 3.1	29.8 ± 5.1	31.7 ± 4.7	33.9 ± 4.4	35.9 ± 4.7	27.8 ± 5.6	30.1 ± 5.0	31.7 ± 4.9	38.8 ± 4.8	33.6 ± 4.7
	P10	25.4	23.18	25.6	27.4	28.5	20.7	23.5	25.5	27.3	26.9
	P25	27.9	26.5	28.1	31.4	31.3	23.6	26.8	28.3	30.8	31.1
	P50	29.8	29.5	31.6	33.9	35.8	28.1	29.9	31.6	33.8	34.2
	P75	32.1	32.7	34.7	37.2	39.4	29.9	32.8	35.0	37.1	37.5
	P90	–	37.05	37.4	39.5	42.0	36.9	36.8	38.8	40.2	38.8
CMJ Power	M ± SD	12.1 ± 0.6	12.0 ± 1.2	12.4 ± 1.0	12.9 ± 0.8	13.3 ± 1.0	11.7 ± 1.1	12.1 ± 1.1	12.4 ± 1.2	12.9 ± 0.9	12.7 ± 0.9
(w/kg)	P10	11.25	10.52	11.13	11.74	11.69	9.94	10.53	11.25	11.5	11.3
	P25	11.74	11.38	11.66	12.46	12.61	10.97	11.39	11.81	12.33	12.30
	P50	12.06	12.10	12.37	13.01	13.38	11.59	12.22	12.34	12.99	12.74
	P75	12.48	12.64	13.14	13.40	14.08	12.14	12.70	13.18	13.51	13.38
	P90	–	13.64	13.75	14.01	14.44	13.82	13.45	14.02	14.14	13.96
CMJ-S Height (cm)	M ± SD	35.3 ± 3.7	35.4 ± 5.8	37.4 ± 5.2	41.3 ± 5.3	43.6 ± 5.5	32.3 ± 5.6	35.9 ± 5.9	37.9 ± 5.4	41.1 ± 6.0	40.4 ± 5.0
	P10	31.2	27.9	30.7	35.0	36.4	24.7	28.8	30.9	32.3	34.1
	P25	31.9	31.2	34.4	38.2	39.3	27.6	31.3	34.7	37.4	37.5
	P50	34.1	34.9	37.2	41.9	42.9	31.8	35.6	37.2	41.2	40.3
	P75	38.6	38.8	40.8	44.9	46.5	36.5	40.0	41.7	45.4	43.4
	P90	–	43.9	42.7	47.1	52.8	39.8	42.8	45.0	47.4	46.6
CMJ-S Power (w/kg)	M ± SD	13.1 ± 0.7	13.1 ± 1.4	13.5 ± 1.0	14.3 ± 0.9	14.7 ± 1.1	12.6 ± 1.2	13.2 ± 1.2	13.6 ± 1.3	14.3 ± 1.1	14.0 ± 0.9
	P10	12.37	11.64	12.16	13.08	13.15	10.85	11.65	12.18	12.63	12.6
	P25	12.55	12.34	13.05	13.81	13.99	11.80	12.30	13.06	13.70	13.34
	P50	12.99	13.14	13.50	14.30	14.73	12.54	13.14	13.51	14.19	14.10
	P75	13.57	13.91	14.20	14.94	15.34	13.43	13.96	14.34	15.08	14.56
	P90	–	14.53	14.70	15.37	16.20	14.38	14.55	15.12	15.54	15.08
Sit and Reach	M ± SD	−2.5 ± 8.1	−1.2 ± 7.5	−0.9 ± 8.1	3.6 ± 9.7	6.0 ± 10.0	−3.0 ± 5.8	−2.0 ± 7.6	0.6 ± 7.7	2.2 ± 10.2	5.9 ± 11.5
(cm)	P10	−11.5	−11.0	−11.6	−10.0	−7.4	−11.4	−11.1	−10.5	−13.4	−13.2
	P25	−10.3	−7.8	−7.2	−4.0	−2.1	−7.4	−8.5	−5.0	−5.1	−3.4
	P50	−2.8	−1.0	−2.0	3.0	5.0	−2.1	−2.4	0.8	2.0	6.8
	P75	3.5	3.9	5.0	11.6	14.0	1.1	4.6	5.3	10.0	15.3
	P90	–	8.1	10.9	15.8	21.1	5.5	7.8	11.2	15.3	23.1

*CMJ, countermovement jump; CMJ-S, countermovement jump with arm swing; MBT, medicine ball throw; SUM HG, sum of right and left handgrip; TT, T-test; V20-m, 20 m speed test; YAPHV, years from age at peak height velocity*.

## Discussion

The aims of this study were: (i) to analyze the morphological and fitness attributes of young male Portuguese basketball players aged 12–16 years and (ii) to establish normative data according to players' chronological ages and the years from APHV.

Anthropometric and physiological attributes are relevant for success in youth basketball players (Fragoso et al., 2015; Johnston et al., 2018; Guimarães et al., 2019a) and should also be considered for the long-term development of athletes (Viru et al., 1999). To the best of our knowledge, this is the first study to establish normative values for morphological and fitness attributes according to the age and maturity status of Portuguese young basketball players.

The results of the present study demonstrated that Portuguese young basketball players had bigger body sizes than equivalent aged national schoolchildren when descriptive comparisons were considered (Santos et al., 2014). Young basketball players are, on average, taller (12 years, +15.9 cm; 13 years, +15.6 cm; 14 years, +13.6 cm; 15 years, +13.4 cm; 16 years, +7.1 cm) and heavier (12 years, +3.4 kg; 13 years, +8.9 kg; 14 years, +6.4 kg; 15 years, +8.6 kg; 16 years, +2.1 kg) than Portuguese schoolchildren of the same chronological age (Santos et al., 2014). Portuguese young basketball players were also found to be taller and heavier than their U-14 Portuguese counterparts who were not selected for regional teams (Guimarães et al., 2019a). These findings are in line with previous research, which showed that body size (i) is an important attribute to discriminate the performance levels in young basketball players (Coelho e Silva et al., 2008; Torres-Unda et al., 2016; Ramos et al., 2020) and (ii) represents the key variable for talent identification and the selection process for elite club teams (Torres-Unda et al., 2013; Ramos et al., 2020). These results offer preliminary support and show the need to create specific norms for young basketball players. The established values for the normal population of equivalent age groups seem to be inadequate to grade these athletes.

Furthermore, the regional players from our study appear to be taller and heavier than Portuguese local basketball players when CA is considered (Arede et al., 2021). However, when players' heights were compared according to YAPHV, the results presented by local basketball players (height: −0.5 YAPHV = 166.7 cm; 0.2 YAPHV = 167.6 cm) became remarkably similar to those shown in our study (height: “0” YAPHV group = 169.2 cm) (Arede et al., 2021). In addition, our U-16 elite regional basketball players seem to be shorter and lighter than the basketball players who were selected to participate in the U-16 Portuguese National Team training camp (height = 180.5 vs. 189.7 cm, body mass = 68.8 vs. 81.1 kg) (Arede et al., 2020). However, it is worth mentioning that U-16 national team players were 3.1 years (early mature) and 2.3 years (average) from PHV (Arede et al., 2020), while our U-16 regional team players were 2.04 years from PHV.

When comparing the norms obtained from the young Portuguese basketball players to those of international elite basketball players of the same CA, Portuguese players showed higher mean height values at age 13 (+5.6 cm), quite similar at ages 14 (−0.8 cm) and 15 (+0.4 cm), but considerably lower at age 16 (−7.9 cm) compared to those of Dutch basketball players (Te Wierike et al., 2014). It should be noted that the sample used in the study with Dutch players was relatively small and that the previous comparison must be analyzed carefully. Nevertheless, Portuguese players, when compared to elite Spanish basketball players at age 13, showed lower mean values both for stature (−7.9 cm) and body mass (−10.6 kg) (Torres-Unda et al., 2013). It is worth mentioning that, in this study, Spanish players were 2.3 years from PHV (Torres-Unda et al., 2013), while Portuguese players were 0.54 years from PHV.

When considering the maturity status of the two samples (Portuguese and Spanish), the results of the comparison became very similar. The Portuguese players were much like their Spanish counterparts (Torres-Unda et al., 2013), showing similar mean values of height (+0.9 cm) and of body mass (−1.9 kg). Taken together, these results reinforce the importance of establishing normative values for young basketball players according to their biological maturity. The typical chronological age norms are particularly important when comparing the observed measures with references by age. These references allow tracking a child's growth and understanding the typology of these changes over time. However, growth is a dynamic process during which it is necessary to distinguish between the morphological variability, typical of a specific growth stage, and that resulting from maturity status differences since fitness improvements induced by growth and maturation changes are surprisingly similar to those induced by training and sports experience. In this regard, normative references according to YAPHV may help to focus on the critical periods (like PHV) in order to evaluate improvements of certain qualities based on biological references and to adjust the strength and conditioning programs to biological demands. Even recognizing that the used methodology may have limitations and the obtained maturation values may not be exactly accurate, as described in *Methods*, this biological characterization will enable distinguishing growth and maturation results from training-induced performance and setting realistic and challenging goals for individual players (early and later maturers) in the medium and the long term. Maturity status, when used properly, can ensure that: (i) late-maturing players who are technically gifted, within their chronological age, are not discriminated against due to their temporary immaturity (Fragoso et al., 2015) and that (ii) early-maturing athletes normally selected for size-related reasons can have a range of motor skill experiences and specific training volumes adjusted to their advance maturity.

Regarding the fitness attributes, our results suggest that the mean values increased with age in speed, COD ability (except from age 12 to 13), MBT distance (except from age 15 to 16), HG strength (except from age 15 to 16), CMJ and CMJ-S height and CMJ and CMJ power (except from age 15 to 16), and also in the sit and reach test. Similar results were observed when the players were divided according to the YAPHV.

Recent research highlighted speed and COD ability as important attributes for basketball youth, both at the individual and team performance levels (Jakovljevic et al., 2012; Ramos et al., 2019, 2020). U-14 basketball players selected for Portuguese regional teams demonstrated significantly better results in the *T*-test than those non-selected (Guimarães et al., 2019a). The U-16 regional players from our study showed similar results in the 20-m sprint test than the 14- to 15-year-old players who participated in the 2016 U-16 Portuguese National Team training camp (Arede et al., 2019). The regional players from our study were almost 0.5 s faster in the *T*-test than the U-16 players who participated in the 2016 U-16 Portuguese National Team training camp (Arede et al., 2019). However, it must be taken into account that the players from our study were 2.04 years from PHV and the players from the mentioned study were 1.13 years from PHV (Arede et al., 2019).

In addition, the 13-year-old players from our study seemed to demonstrate better results in the 20-m sprint test and the *T*-test than did the U-13 local basketball players from a recent study (Arede et al., 2021). When the speed and COD ability results are compared according to the YAPHV, the differences between the values reported in both studies disappear, and regional players show quite similar results in speed (20-m test: “0” YAPHV group = 3.34 s) and COD ability (*T*-test: “0” YAPHV group = 10.31 s) when compared to local basketball players (20-m test: 0.2 YAPHV = 3.3 s; *T*-test: 0.2 YAPHV = 10.3 s) (Arede et al., 2021). When considering international basketball, the descriptive comparison revealed that young Portuguese basketball players were faster in the 20-m sprint test (12 years, −0.35 s; 14 years, −0.54 s) and in the *T*-test (12 years, −1.74 s; 14 years, −0.62 s) than elite Serbian basketball players of 12 and 14 years (Jakovljevic et al., 2012). However, when compared to Spanish elite basketball players at age 13, Portuguese players showed worse performances in the 20-m sprint test than their Spanish counterparts (Jakovljevic et al., 2012; Torres-Unda et al., 2013, 2016). The observed differences between Portuguese and Spanish 13-year-old basketball players may be explained by the more advanced maturity status of the Spanish players (Torres-Unda et al., 2013). These findings once again emphasize the importance of establishing normative values for physical qualities according to maturity development, in particular for speed and COD ability, described as important attributes in young players' basketball performance and considered as discriminant factors in a team's classification at a national tournament for U-14 regional selection teams (Ramos et al., 2020).

Upper body strength, evaluated by the MBT and HG strength test, was confirmed as an influencing factor on youth basketball performance (Chaouachi et al., 2009; Ramos et al., 2019, 2020). Recent studies reported that U-14 elite basketball players from higher-ranked teams demonstrated better results in the 2-kg MBT than players from the lower-ranked teams (Ramos et al., 2020), and HG strength was identified as one of the predictors of youth basketball players' individual performance (i.e., Performance Index Rating) (Ramos et al., 2019). When compared to other elite Portuguese players aged 12–13.99 years (Coelho e Silva et al., 2010) and 14–15.99 years (Coelho e Silva et al., 2008), the sport practitioners from our sample demonstrated similar results in the HG strength test. Furthermore, the U-16 regional players from our study threw the 2-kg medicine ball (MBT) over a longer distance than did the 14- to 15-year-old players who participated in the 2016 U-16 Portuguese National Team training camp (Arede et al., 2019). It is worth mentioning that the players from our study were 0.9 years ahead of PHV when compared to the players selected for the U-16 Portuguese National Team training camp (Arede et al., 2019), which could have influenced the results. Several studies highlighted the relevance of maturity status for basketball players' upper body strength and power (evaluated by the 2-kg MBT), where more mature players show higher upper body power than less matured players (Coelho e Silva et al., 2008, 2010; Arede et al., 2019).

Jumping ability was singled out as a relevant attribute for basketball performance since vertical jumps are among the most prevalent actions performed by basketball players in both defense (e.g., rebounding and blocking) and offense (e.g., shooting and rebounding) (Ostojic et al., 2006). A previous study of 125 young Australian basketball players revealed significant differences in the vertical jump among players of different skill levels (Hoare, 2000). The best players tended to jump higher when compared to other players (Hoare, 2000). Our results for the CMJ heights of 12- and 13-year-old Portuguese basketball players were similar (12 years, +1.5 cm; 13 years, −1.5 cm) to those presented by Coelho e Silva and colleagues, while our 14- and 15-year-old basketball players jumped less, 1.8 and 4 cm, respectively, than the Portuguese national level players of the same chronological age (Coelho e Silva et al., 2010). These results can be explained by the difference in the players' competitive levels. In our study, the sample consisted of the best Portuguese regional players, while Coelho e Silva et al. (2010) analyzed players from five youth clubs who played at a district level. Another explanation may be related to the time lapse between the data collections and the variations in body size and physical fitness levels due to environmental influences and the trend of increasing body size and faster growth rate described in the special literature (Malina, 1991). Furthermore, our U-16 regional players showed similar results in the CMJ and CMJ-S to the U-16 prepubertal players who participated in the 2016 Portuguese National Team training camp (Arede et al., 2019). These results are interesting since, in our study, the players were 2.04 years to the PHV, while the U-16 prepubertal players were 0.31 years from the PHV, which suggests that the national-level players, who were less mature, may still be improving their jumping ability and will surpass the results presented by the regional players introduced in this study.

Regarding the comparison with international basketball players, our results demonstrated that young Portuguese basketball players had a similar jumping ability to Greek basketball players of the same chronological age (13 years, −0.1 cm; 14 years, −1.2 cm; 15 years, +0.3 cm; 16 years, +0.5 cm) (Kellis et al., 1999). It is worth noting that there is almost a 20-year difference between the two studies, and the differences in strength and power between generations can influence the previous comparison (Kellis et al., 1999). However, when compared to Spanish basketball players, the differences in the jumping height between the two samples increased considerably. On average, 13-year-old Portuguese basketball players jumped less 11.3 cm (Torres-Unda et al., 2013) and 7.6 cm (Torres-Unda et al., 2016) in the CMJ-S test than did elite Spanish players of the same chronological age. An individual's growth and abilities change rapidly during adolescence and can adopt different individual growth ratings over time (Moran et al., 2020). This reinforces the importance of an athlete's categorization in maturity-related groups (i.e., pre-PHV, circa-PHV, and post-PHV) in order to better understand and, if possible, anticipate their physical changes. Children during pre-PHV, due to their neuromuscular plasticity and increased tissue pliability (Eston et al., 2003), can typically recover more quickly from fatigue-inducing resistance training sessions (Faigenbaum et al., 2008) and, consequently, can stand shorter rest intervals during their resistance training programs designed to improve muscle strength and motor control. Furthermore, some young athletes may show “adolescence awkwardness,” which typically occurs 6 months before PHV, which can possibly explain the temporary motor skill disruption, such as coordination, movement economy, and, consequently, sports performance. This developmental phenomenon is particularly evident in athletes with an early-onset adolescent growth spurt (Philippaerts et al., 2006; Quatman-Yates et al., 2012), making maturity categorization a positive tool to prevent and explain motor skill irregularities. Additionally, athletes circa-PHV should avoid excessive loadings due to their skeletal fragility and lower motor control patterns, and children during post-PHV should participate in hypertrophy-based resistance training, taking advantage of their body composition growth due to their higher anabolic hormone concentrations and knowledge about resistance training (Lloyd et al., 2014).

To better understand the reasons that lead to the high level of performance in adulthood, it is important to analyze the pathways of young players, aiming to identify the attributes that distinguish young players who were selected at each stage of their long-term development. In this sense, comparing the realities and experiences of different European countries seems quite interesting and useful.

In summary, this study presents specific normative values of Portuguese young basketball players according to their chronological age and maturity status. Having in mind the recent publication of Santos et al. (2014), our findings suggest that basketball players had a bigger body size (between 15.9 cm at 12 years and 7.1 cm at 16 years) than the normal Portuguese population, highlighting the importance of using athlete-specific norms. Moreover, maturity-related growth differences among male adolescent athletes are well-documented in available literature (Malina et al., 2004). During a growth spurt, male athletes of the same chronological age can differ by as much as four or five biological years. This biological difference can result in great physical fitness advantages for early-maturing boys (Coelho e Silva et al., 2008, 2010; Torres-Unda et al., 2013, 2016; Arede et al., 2019, 2021; Ramos et al., 2019, 2020), supporting the need for establishing normative data according to maturity status. Therefore, the identification of performance-related attributes of young basketball players must always consider the different stages of development for each capacity, the individual growth rate, and the average speed of acquisition considering two subsequent periods of development of a particular attribute.

## Practical Applications

The present study provides a first for age- and maturity-specific morphological and fitness reference standards for young Portuguese basketball players aged 12–16 years. This characterization is especially useful when used to quantify and control athletes' performance over time. Even though recognizing that the regression equation of Mirwald et al. (2002) may have some limitations, it can also be of great use if complemented with other longitudinal measurements, like stature and leg length. These morphological and fitness references will help: (i) to distinguish the growth and maturation results from training-induced performance; (ii) to detect physical and fitness weaknesses and attributes during growth and contribute to their correction; (iii) to fix realistic and challenging goals for each player in the medium and the long term; (iv) to identify the adolescent awkwardness particularly evident in athletes with an early-onset adolescent growth spurt; (v) to assure that late-maturing players who are technically gifted are not discriminated due to their temporary immaturity; (vi) to guarantee that early-maturing athletes, normally selected for size-related reasons, can have a range of motor skill experiences and specific training volumes adjusted to their advance maturity; (vii) to prescribe shorter rest intervals during resistance training programs to pre-PHV children; (viii) to avoid excessive loadings to circa-PHV children due to their skeletal fragility and lower motor control patterns; and (viiii) to prescribe hypertrophy-based resistance training to children during post-PHV due to their higher anabolic hormone concentrations and knowledge about resistance training.

Therefore, the references presented in this study cannot be a training tool to be used at a precise moment as an individual recommendation, *per se*, but instead, they can be of help when coaches focus on the reliability of frequent measurements of growth (growth tracking) to capture the individual reality over a period of time.

## Data Availability Statement

The raw data supporting the conclusions of this article will be made available by the authors, without undue reservation.

## Ethics Statement

This study was reviewed and approved by the Ethics Committee of the Faculty of Physical Education and Sport—Universidade Lusófona and was performed according to the Helsinki Declaration. Written informed consent was obtained from all participants for their participation in this study.

## Author Contributions

All authors listed have made a substantial, direct and intellectual contribution to the work, and approved it for publication.

## Conflict of Interest

The authors declare that the research was conducted in the absence of any commercial or financial relationships that could be construed as a potential conflict of interest.
